# Association of ACE2 genetic polymorphisms with hypertension-related target organ damages in south Xinjiang

**DOI:** 10.1038/s41440-018-0166-6

**Published:** 2018-12-12

**Authors:** Yi Luo, Cheng Liu, Tianwang Guan, Yanfang Li, Yanxian Lai, Fang Li, Haiyan Zhao, Tutiguli Maimaiti, Abudurexiti Zeyaweiding

**Affiliations:** 1 Department of Cardiology, Guangzhou First People’s Hospital, Medical School, South China University of Technology, 510180 Guangzhou, China; 2 Department of Cardiology, Guangzhou First People’s Hospital, Guangzhou Medical University, 510180 Guangzhou, China; 3 Department of Cardiology, Shufu People’s Hospital, Xinjiang Uygur Autonomous Region (XUAR), 844100 Kashgar Region, China

**Keywords:** Association, ACE2 polymorphism, Essential hypertension, Target organ damage, South Xinjiang

## Abstract

Essential hypertension (EH) is a principal contributing factor in worldwide cardiovascular disease mortality. Although interventions that minimize environmental risk factors for EH are associated with reduced cardiovascular disease, such approaches are limited for individuals with high genetic EH risk. In this study, we investigated possible associations between ACE2 polymorphisms and hypertension-related target organ damages in south Xinjiang, China. Four hundred and two hypertensive patients were enrolled as study participants in an EH group, and 233 normotensive individuals were enrolled as control subjects. Participants were recruited from the south Xinjiang region. Fourteen ACE2 polymorphisms were genotyped by matrix-assisted laser desorption ionization time-of-flight mass spectrometry. Risk genotypes of rs2074192 (TT+CT, OR = 1.72, 95% CI: 1.17–2.53), rs2106809 (TT, OR = 1.71, 95% CI: 1.13–2.58), rs4240157 (CC+CT, OR = 1.99, 95% CI: 1.17–3.41), rs4646155 (TT+CT, OR = 1.94, 95% CI: 1.06–3.54), rs4646188 (TT+CT, OR = 3.25, 95% CI: 1.95–5.41), rs4830542 (CC+CT, OR = 1.88, 95% CI: 1.10–3.23), and rs879922 (CC+CG, OR = 4.86, 95% CI: 2.74–8.64) were associated with EH. Hypertensive patients carrying the control genotype of rs2074192 (CC, OR = 2.37, 95% CI: 1.28–4.39) were associated with CAS ≥50%, while those carrying a high-EH-risk genotype of rs4240157 (OR = 2.62, 95% CI: 1.24–5.54), rs4646155 (OR = 2.44, 95% CI: 1.16–5.10), or rs4830542 (CC+CT, OR = 2.20, 95% CI: 1.03–4.69) were associated with atrial fibrillation (AF), larger left atrial diameter, and higher levels of renin–angiotensin–aldosterone system (RAAS) activation (renin and angiotensin I/II). In conclusion, the ACE2 variant rs2074192 was associated with EH and EH with CAS ≥50%, while 3 ACE2 variants (rs4240157, rs4646155, and rs4830542) were associated with EH- and hypertension-related AF and left atrial remodeling in south Xinjiang, China.

## Introduction

Essential hypertension (EH) is attributable to both genetic and environmental factors and is a major contributor to worldwide cardiovascular disease (CVD) mortality [1]. A recent prospective cohort study indicates that the prevalence of hypertension in China overall is as high as 32.5% [2], and the EH control rate for Chinese patients overall is only 4.2%, significantly lower than the 30–55% rate reported for Western patients [3, 4]. Under these circumstances, addressing the challenge of preventing and treating EH in China should focus on decreasing prevalent risk factors that potentially affect all members of society [5, 6]. Increasingly, comprehensive management of multiple modifiable risk factors (e.g., unhealthy diet, lack of physical activity, smoking, obesity, dyslipidemia, abnormal glucose metabolism, and obstructive sleep apnea) has been associated with lower blood pressure and lower incidence of EH-related cardiovascular events, including arteriosclerosis cardiovascular disease (ASCVD), atrial fibrillation (AF), heart failure, and other events [3, 7].

By contrast, nonbehavioral/environmental/lifestyle-associated EH risk factors, including male gender, increasing age, and, particularly, genetic factors, will require different interventional approaches. Genetic background notwithstanding, the cardiovascular benefits from management of those reversible cardiovascular risk factors could be weakened or offset by high genetic risk because it is impossible to modify the genetic structure [8, 9]. The time of intervention (which is much more important than intervention itself sometimes, as described above) is the deciding factor affecting the final outcome, so clinical assessment of genetic background is a feasible way to find the optimal intervention time for the population at different levels of EH genetic risk.

Studies to identify associated disease risk gene variants in the Chinese population are essential to begin developing individualized precision approaches to prevent EH. Numerous candidate genes have been implicated in susceptibility to EH. In recent years, genes of the renin–angiotensin–aldosterone system (RAAS) have received a good deal of attention. Angiotensin-converting enzyme 2 (ACE2) is a key RAAS enzyme and a new target for prevention and treatment of EH [10]. ACE2 maps to chromosome Xp22, spans 39.98 kb of genomic DNA, and contains 20 introns and 18 exons. The ACE2 gene encodes a type I membrane-bounded glycoprotein composed by 805 amino acids. Functional domains include a C-terminal transmembrane anchoring region, N-terminal signal peptide region, and an HEXXH zinc-binding metalloprotease motif. The ACE2 gene exhibits a high degree of genetic polymorphism. Unlike the association of ACE2 single-nucleotide polymorphisms (SNPs) with EH in Europeans (rs2074192, rs233575, and rs2158083) [11], Australians (rs1978124 and rs2285666) [12], Indians (rs2285666) [13] and Koreans (rs1514282 and rs1514283) [14], ACE2 polymorphisms associated with EH in the Chinese population display considerable diversity between inhabitants of southern vs. northern China, Han vs. non-Han nationality, and between females and males. A meta-analysis of ACE2 gene polymorphism and EH association confirmed rs2285666 and rs2106809 were correlated with hypertension risk in persons of Han descent [15]. In Han people of northern China, males carrying the G allele and females of genotype GG of rs2285666 had increased risk of EH, and the male G allele frequency of rs1978124 was similar to that of rs2285666 [16]. In the northwestern region, rs2285666 was not associated with EH in Han Chinese but was related to hypertension risk among Dongxiang people [17]. In central China, no significant relationship was found between rs2285666 or rs2106809 and EH in persons of Han descent [18]. In southern China, Han females with CC+CT genotypes for rs2106809 had higher EH risk [19]. SNPs rs4646155 and rs879922 were not associated with EH among the Han [20]. SNP rs2285666 was linked to EH in Li people [21].

Not all ACE2 variants are associated with risk of EH, and among risk-associated ACE2 polymorphic loci, higher EH risk is also linked to geographical, ethnic, and gender-related factors. In this study, we investigated possible associations between ACE2 gene variation and hypertension-related target organ damage in south Xinjiang, China.

## Materials and methods

### Study participants

This study was reviewed and approved by the Ethics Committee of Guangzhou First People’s Hospital, South China University of Technology. A total of 402 consecutive patients with EH and 233 normotensive subjects from the south Xinjiang region were enrolled in the study from 2012 to 2017. All participants were long residents of the region and were from multigeneration resident families. All participants shared the same high-salt, high-fat diet. The newly hypertensive patients were diagnosed according to the criteria of the 1999 World Health Organization/International Society of Hypertension (WHO/ISH) as follows: [1] systolic blood pressure (SBP) ≥140 mm Hg and/or diastolic blood pressure (DBP) ≥90 mm Hg; [2] diagnosed with EH for the first time and did not receive any antihypertensive treatment; and [3] family history of high blood pressure. Exclusion criteria: [1] secondary hypertension, due to conditions such as renal parenchymal hypertension, renal vascular hypertension (renal artery stenosis), endocrine diseases such as primary aldosteronism, pheochromocytoma, Cushing syndrome, and hyperthyroidism; [2] white coat hypertension; and [3] any other diseases or drugs that may affect blood pressure. The healthy normotensive subjects were defined as those without a history of hypertension and with blood pressure measurements of SBP < 130 mm Hg and DBP < 85 mm Hg [22]. Blood pressure was measured in the seated position after 10 min of rest using a mercury sphygmomanometer by experienced and certified examiners, and it was measured in the brachial artery 3 times at 5-min intervals in at least two separate visits to the health-care office. The mean of the last two measurements per visit was recorded as representative of clinical BP. Bilateral carotid duplex scanning was performed on admission to the study. A comparison of the baseline characteristics is shown in Table 1.

**Table1:** Baseline characteristics of study participants

	Normotensive	Hypertensive	*P* value
*N*	233	402	—
Han:Uygur	116:117	222:180	0.186
Male:Female	93:140	166:236	0.733
Age (Years)	57.7 ± 12.6	58.8 ± 10.9	0.298
Smoking (%)	43 (18.3)	82 (20.4)	0.553
Drinking (%)	40 (17.2)	83 (20.6)	0.285
SBP (mm Hg)	116.2 ± 9.5	155.8 ± 13.3	<0.001
DBP (mm Hg)	73.9 ± 7.4	85.5 ± 13.5	<0.001
BMI (Kg/m^2^)	23.5 ± 4.6	26.1 ± 4.1	<0.001
TRIG (mmol/L)	1.29 ± 0.95	1.35 ± 0.63	0.350
CHOL (mmol/L)	4.56 ± 1.12	4.64 ± 1.21	0.370
HDL-C (mmol/L)	1.24 ± 0.41	1.20 ± 0.28	0.088
LDL-C (mmol/L)	2.38 ± 0.76	2.86 ± 0.80	<0.001
Lp(a) (g/L)	0.24 ± 0.19	0.24 ± 0.21	0.621
ApoA1/ApoB	1.32 ± 0.42	1.38 ± 0.55	0.105
FBG (mmol/L)	5.90 ± 1.89	6.02 ± 1.92	0.422
Cr (μmol/L)	74.5 ± 24.0	77.7 ± 23.2	0.103
BUN (mmol/L)	5.20 ± 1.99	5.28 ± 1.72	0.614
UA (μmol/L)	308.5 ± 135.7	355.4 ± 131.7	<0.001
ALT (U/L)	25.3 ± 15.0	26.8 ± 22.1	0.347
AST (U/L)	23.3 ± 12.2	23.3 ± 13.7	0.965
Alb (g/L)	37.2 ± 4.4	37.6 ± 6.0	0.412
Na^+^ (mmol/L)	138.1 ± 3.4	140.3 ± 4.3	<0.001
Ca^2+^ (mmol/L)	2.19 ± 0.11	2.20 ± 0.12	0.096
K^+^ (mmol/L)	4.03 ± 0.41	3.97 ± 0.49	0.097
Mg^2+^ (mmol/L)	1.01 ± 0.18	1.00 ± 0.18	0.511
HsCRP (mg/L)	10.8 ± 18.9	14.9 ± 24.1	0.018
ACE (U/L)	36.6 ± 19.8	42.0 ± 25.7	0.006
Renin (pg/mL)	24.5 ± 28.1	34.7 ± 33.3	<0.001
Ang I (ng/L)	2.07 ± 1.44	2.70 ± 1.77	<0.001
Ang II (ng/L)	62.7 ± 93.9	90.6 ± 123.9	0.001
ALD (ng/L)	187.2 ± 114.4	247.0 ± 133.0	<0.001

### Carotid and cardiac ultrasonography

Bilateral carotid and cardiac ultrasonic scanning was performed on admission to the study. The near and far walls of the bilateral common carotid artery, bifurcations, and 1 cm of the internal and external carotid arteries were scanned for the presence of carotid arteriosclerosis stenosis (CAS) ≥50% (recorded as the average of two independent experienced physician measurements according to the measurement of the degree of stenosis used in the North American Symptomatic Carotid Endarterectomy Trial [23]) with a 3/9 MHz ultrawideband linear array transducer (iU22, Philips, NL). Left atrial diameter (LAD) was measured using M-mode or two-dimensional echocardiography in the parasternal long-axis view at the end-ventricular systole with a 1.7/3.4 MHz linear array transducer (Vivid 7, GE Healthcare, USA) over 4 cardiac cycles according to recommendations for chamber quantification from the American Society of Echocardiography [24].

### Genotyping assay

Genomic DNA was extracted from whole blood using the Maxwell RSC Whole Blood DNA Kit (Promega, Madison, WI), quantified using a NanoDrop-1000 (ThermoFisher, Waltham, MA) and diluted to 10 ng/μL concentration. Fourteen ACE2 SNPs (rs1978124, rs2048683, rs2074192, rs2106809, rs2285666, rs233575, rs4240157, rs4646142, rs4646155, rs4646156, rs4646188, rs4830542, rs6632677, and rs879922) were identified based on existing literature and human genome sequence databases. Primers for ACE2 SNPs were designed based upon sequence information from GenBank using Primer 5.0 (Whitehead Institute Cambridge, MA, USA) and Operon’s Oligo software 7.60 (OperonTechnologies Inc., Alameda, California, USA). Primers are shown in Supplementary Table 1. ACE2 SNPs were analyzed using the Sequenom MassARRAY system according to previously described methods [25]. Genotyping accuracy was determined by genotype concordance between duplicate samples and was 100% for each SNP.

### Statistical analysis

Because ACE2 is located on the X chromosome, Hardy–Weinberg equilibrium was assessed only for females, as shown in Supplementary Table 2. Analysis was performed using SPSS version 20 (SPSS, Chicago, IL) and PASS version 15 (Statistical Solutions Ltd, Cork, Ireland). Categorical variables (nationality, gender, smoking, drinking, EH, and EH complicated with CAS ≥ 50% or AF) are presented as frequencies. The relationship between each ACE2 SNP and those categorical variables was assessed using the chi-square test. The odds ratio (OR) between the control genotype and the high-hypertensive-risk genotype for each ACE2 SNP for categorical variables was evaluated using binary logistic regression. Considering the possible false-positive risk to the final result, Bonferroni adjustment was applied to adjust the *P* value obtained in multilogit regression. Continuous variables (age, SBP, DBP, body mass index (BMI), blood biochemical index, and LAD) are presented as the mean ± SD. Significant differences for continuous variables were analyzed by two-way analysis of variance (ANOVA), one-way ANOVA, or the independent-sample*t* test, according to our research design. The least significant difference test was further used to assess differences between two subgroups after variance analysis, to show distinct differences with homogeneous variance, while the Games–Howell test was used for heterogeneous variance. A *P* value <0.05 was considered statistically significant. All probabilities are two-tailed.

## Result

### Characteristics of the study participants

As shown in Table 1, hypertensive and normotensive subjects showed significant differences in SBP, DBP, BMI, low-density lipoprotein cholesterol (LDL-C), serum uric acid, serum sodium, high-sensitivity C-reactive protein, and the activation of RAAS (*P* < 0.001 or *P* = 0.001) but not in nationality, age, gender, smoking, drinking, hypertriglyceridemia, hypercholesterolemia, high-density lipoprotein cholesterol (HDL-C), lipoprotein A), fasting blood glucose, renal function (creatinine, blood urea nitrogen), liver function (alanine transaminase, aspartate aminotransferase, albumin), or blood electrolytes (calcium, potassium, and magnesium) (all *P* > 0.05).

### Association of ACE2 SNPs with EH

As shown in Table 2, ACE2 SNPs rs2074192 (*P* = 0.006), rs2106809 (*P* = 0.012), rs4240157 (*P* = 0.012), rs4646155 (*P* = 0.030), rs4646188 (*P* < 0.001), rs4830542 (*P* = 0.020), and rs879922 (*P* < 0.001) were significantly associated with EH except rs1978124, rs2048683, rs2285666, rs233575, rs4646142, rs4646156, and rs6632677 (all *P* > 0.05, see Supplementary Table 3).

**Table 2:** Association of ACE2 SNPs with EH in participants

ACE2 SNPs		Normotensive (*N* (%))	Hypertensive (*N* (%))	OR (95% CI)^a^	*P* value^a^
rs2074192	CC	118 (50.6)	162 (40.3)	1.00	
	TT+CT	115 (59.4)	240 (59.7)	1.72 (1.17–2.53)	0.006
rs2106809	CC+CT	121 (51.9)	175 (43.5)	1.00	
	TT	112 (48.1)	227 (56.5)	1.71 (1.13–2.58)	0.012
rs4240157	CC+CT	43 (18.5)	92 (22.9)	1.99 (1.17–3.41)	0.012
	TT	190 (81.5)	310 (77.1)	1.00	
rs4646155	CC	211 (90.6)	334 (83.1)	1.00	
	TT+CT	22 (9.4)	68 (16.9)	1.94 (1.06–3.54)	0.030
rs4646188	CC	74 (31.8)	85 (21.1)	1.00	
	TT+CT	159 (68.2)	317 (78.9)	3.25 (1.95–5.41)	<0.001
rs4830542	CC+CT	43 (18.5)	90 (22.4)	1.88 (1.10–3.23)	0.020
	TT	190 (81.5)	312 (77.6)	1.00	
rs879922	CC+CG	40 (17.2)	131 (32.6)	4.86 (2.74–8.64)	<0.001
	GG	193 (82.8)	271 (67.4)	1.00	

^a^After adjustment for nationality, gender, age, smoking, BMI, TRIG, LDL-C, HDL-C, Lp(a), FBS, UA, HsCRP, and Ang II

### Association of ACE2 SNPs with EH complicated by CAS ≥ 50%

As shown in Table 3, ACE2 SNPs rs2074192 (*P* = 0.006), rs2285666 (*P* = 0.024), and rs4646142 (*P* = 0.023) were associated with EH complicated by CAS ≥ 50%.

**Table 3:** Association of ACE2 SNPs with EH complicated by CAS ≥ 50% in hypertensive participants

ACE2 SNPs		Non-CAS ≥ 50% (*N* (%))	CAS ≥ 50% (*N* (%))	OR (95% CI)^a^	*P* value^a^
rs2074192	CC	126 (37.2)	36 (57.1)	2.37 (1.28–4.39)	0.006
	TT+CT	213 (62.8)	27 (42.9)	1.00	
rs2285666	CC	131 (38.6)	15 (23.8)	1.00	
	TT+CT	208 (61.4)	48 (76.2)	2.28 (1.11–4.65)	0.024
rs4646142	CC+CG	206 (60.8)	48 (76.2)	2.28 (1.12–4.66)	0.023
	GG	133 (39.2)	15 (23.8)	1.00	

^a^After adjustment for nationality, gender, age, smoking, BMI, LDL-C, HDL-C, FBS, UA, HsCRP, and Ang II

### Association of ACE2 SNPs with EH-related left atrial enlargement (LAE)

As shown in Table 4, no significant difference was observed between hypertensive and normotensive participants in LAD size (all *P* > 0.05), while significant difference were observed between the high-EH-risk and control genotypes of the EH-risk-related ACE2 SNPs rs4240157 (*P* = 0.007), rs4646155 (*P* = 0.029), and rs4830542 (*P* = 0.003).

**Table 4:** Association of ACE2 SNPs with EH-related left atrial enlargement in participants

ACE2 SNPs		LAD (cm)		
		Normotensive	Hypertensive	*P* value
rs2074192	CC	2.77 ± 0.26	2.76 ± 0.41	0.834
	TT+CT	2.73 ± 0.33	2.81 ± 0.46	0.056
	*P* value	0.332	0.229	
rs2106809	CC+CT	2.74 ± 0.34	2.83 ± 0.37	0.054
	TT	2.76 ± 0.26	2.77 ± 0.49	0.881
	*P* value	0.582	0.186	
rs2285666	CC	2.74 ± 0.33	2.86 ± 0.51	0.040
	TT+CT	2.76 ± 0.28	2.76 ± 0.40	0.976
	*P* value	0.771	0.047	
rs4240157	CC+CT	2.72 ± 0.27	2.93 ± 0.40	<0.001
	TT	2.76 ± 0.31	2.75 ± 0.45	0.861
	*P* value	0.404	0.001	
rs4646142	CC+CG	2.77 ± 0.27	2.76 ± 0.40	0.706
	GG	2.72 ± 0.34	2.86 ± 0.51	0.016
	*P* value	0.212	0.044	
rs4646155	CC	2.75 ± 0.31	2.77 ± 0.45	0.551
	TT+CT	2.75 ± 0.24	2.90 ± 0.42	0.032
	*P* value	0.989	0.023	
rs4646188	CC	2.79 ± 0.32	2.78 ± 0.37	0.802
	TT+CT	2.73 ± 0.29	2.80 ± 0.46	0.059
	*P* value	0.154	0.693	
rs4830542	CC+CT	2.72 ± 0.27	2.94 ± 0.39	<0.001
	TT	2.76 ± 0.31	2.75 ± 0.45	0.779
	*P* value	0.404	<0.001	
rs879922	CC+CG	2.69 ± 0.26	2.80 ± 0.45	0.051
	GG	2.76 ± 0.31	2.79 ± 0.44	0.462
	*P* value	0.143	0.823	

### Association of ACE2 SNPs with EH complicated by AF

As shown in Table 5, ACE2 SNPs rs2285666 (*P* = 0.039), rs4240157 (*P* = 0.011), s4646142 (P = 0.014), rs4646155 (*P* = 0.018), and rs4830542 (*P* = 0.042) were associated with EH complicated by AF.

**Table 5:** Association of ACE2 SNPs with EH complicated by AF in hypertensive participants

ACE2 SNPs		Non-AF (*N* (%))	AF (*N* (%))	OR (95% CI)^a^	*P* value^a^
rs2285666	CC	109 (33.4)	37 (48.7)	1.95 (1.03–3.66)	0.039
	TT+CT	217 (66.6)	39 (51.3)	1.00	
rs4240157	CC+CT	65 (19.9)	27 (35.5)	2.62 (1.24–5.54)	0.011
	TT	261 (80.1)	49 (64.5)	1.00	
rs4646142	CC+CG	215 (66.0)	39 (51.3)	1.00	
	GG	111 (34.0)	37 (48.7)	2.36 (1.19–4.66)	0.014
rs4646155	CC	276 (84.7)	58 (76.3)	1.00	
	TT+CT	50 (15.3)	18 (23.7)	2.44 (1.16–5.10)	0.018
rs4830542	CC+CT	65 (19.9)	25 (32.9)	2.20 (1.03–4.69)	0.042
	TT	261 (80.1)	51 (67.1)	1.00	

^a^After adjustment for nationality, gender, age, smoking, BMI, SBP, DBP, LDL-C, HDL-C, FBS, UA, HsCRP, Ang II, and LAD

### Association of ACE2 SNPs with RAAS activation

As shown in Table 6, significant differences were observed between hypertensive and normotensive participants in the levels of ACE, renin, angiotensin I/II (ANG I/ II), and aldosterone (ALD) (all *P* < 0.05). Significant differences were also observed between the high-EH-risk and control genotypes of the EH-risk-related ACE2 SNPs in the levels of ACE (rs2106809 (*P* = 0.008), rs4646188 (*P* = 0.032), rs4830542 (*P* = 0.48), and rs879922 (*P* = 0.005)), renin (rs4240157 (*P* = 0.016), rs4646155 (*P* = 0.009), rs4830542 (*P* = 0.022), and rs879922 (*P* < 0.001)), ANG I (rs4646188 (*P* = 0.028) and rs879922 (*P* = 0.005)), and ANG II (rs4240157 (*P* = 0.001), rs4830542 (*P* = 0.001), and rs879922 (*P* < 0.001)) but not ALD (all *P* > 0.05).

**Table 6:** Association of ACE2 SNPs with the RAAS activity in participants

ACE2 SNPs		ACE (U/L)			Renin (pg/mL)			Ang I (ng/L)		
		Normotensive	Hypertensive	*P* value	Normotensive	Hypertensive	*P* value	Normotensive	Hypertensive	*P* value
rs2074192	CC	35.3 ± 18.6	41.9 ± 31.6	0.044	25.8 ± 28.8	34.9 ± 35.4	0.022	2.00 ± 1.41	2.78 ± 1.83	<0.001
	TT+CT	35.9 ± 19.9	42.1 ± 20.9	0.009	23.3 ± 27.3	34.5 ± 31.9	0.001	2.14 ± 1.47	2.64 ± 1.73	0.008
	*P* value	0.791	0.931		0.487	0.894		0.445	0.452	
rs2106809	CC+CT	34.2 ± 20.4	38.5 ± 22.3	0.094	24.9 ± 31.0	30.4 ± 33.3	0.151	2.23 ± 1.59	2.45 ± 1.69	0.261
	TT	37.1 ± 17.9	44.7 ± 27.7	0.009	24.1 ± 24.7	37.9 ± 33.1	<0.001	1.89 ± 1.25	2.89 ± 1.81	<0.001
	*P* value	0.257	0.016		0.825	0.026		0.065	0.015	
rs4240157	CC+CT	38.9 ± 13.8	45.1 ± 16.6	0.024	26.2 ± 24.3	42.4 ± 34.2	0.002	1.90 ± 1.39	3.08 ± 1.76	<0.001
	TT	34.9 ± 20.2	41.1 ± 27.8	0.008	24.2 ± 28.9	32.4 ± 32.8	0.005	2.10 ± 1.45	2.58 ± 1.76	0.001
	*P* value	0.121	0.187		0.676	0.011		0.412	0.018	
rs4646155	CC	35.3 ± 18.1	41.7 ± 25.5	0.002	25.2 ± 28.2	36.4 ± 34.6	<0.001	2.05 ± 1.43	2.66 ± 1.75	<0.001
	TT+CT	38.1 ± 28.1	43.3 ± 26.9	0.443	18.4 ± 26.1	26.1 ± 24.4	0.208	2.14 ± 1.59	2.88 ± 1.85	0.097
	*P* value	0.654	0.657		0.279	0.020		0.795	0.355	
rs4646188	CC	37.6 ± 21.9	46.7 ± 38.7	0.078	19.5 ± 22.2	32.6 ± 31.4	0.002	2.09 ± 1.52	2.21 ± 1.61	0.629
	TT+CT	34.7 ± 17.8	40.7 ± 20.8	0.002	26.9 ± 30.2	35.2 ± 33.8	0.009	2.06 ± 1.41	2.83 ± 1.79	<0.001
	*P* value	0.310	0.059		0.058	0.519		0.864	0.004	
rs4830542	CC+CT	38.9 ± 13.8	45.7 ± 16.2	0.013	26.2 ± 24.3	42.1 ± 34.5	0.003	1.90 ± 1.39	3.03 ± 1.75	<0.001
	TT	34.9 ± 20.2	40.9 ± 27.7	0.009	24.2 ± 28.9	32.5 ± 32.7	0.004	2.10 ± 1.45	2.60 ± 1.76	0.001
	*P* value	0.121	0.119		0.676	0.016		0.412	0.041	
rs879922	CC+CG	39.3 ± 14.2	46.4 ± 16.8	0.010	26.3 ± 25.2	47.1 ± 36.7	<0.001	1.96 ± 1.43	3.12 ± 1.83	<0.001
	GG	34.8 ± 20.1	39.9 ± 28.8	0.036	24.2 ± 28.7	28.6 ± 29.8	0.106	2.09 ± 1.45	2.49 ± 1.70	0.006
	*P* value	0.099	0.018		0.657	<0.001		0.559	0.001	

ACE2 SNPs		Ang II (ng/L)			ALD (ng/L)		
		Normotensive	Hypertensive	*P* value	Normotensive	Hypertensive	*P* value
rs2074192	CC	82.5 ± 95.3	95.5 ± 103.1	0.281	202.0 ± 130.4	248.3 ± 126.9	0.003
	TT+CT	75.2 ± 63.6	97.0 ± 119.0	0.047	192.8 ± 93.5	239.0 ± 118.5	<0.001
	*P* value	0.538	0.896		0.537	0.460	
rs2106809	CC+CT	85.2 ± 99.2	79.8 ± 109.9	0.667	190.4 ± 91.1	235.9 ± 122.0	<0.001
	TT	72.1 ± 77.8	109.2 ± 113.5	<0.001	205.1 ± 133.6	248.1 ± 121.8	0.003
	*P* value	0.260	0.009		0.322	0.319	
rs4240157	CC+CT	81.7 ± 73.4	133.5 ± 124.2	0.003	199.3 ± 174.6	258.2 ± 117.8	0.023
	TT	78.3 ± 93.0	85.4 ± 106.9	0.430	197.0 ± 95.1	238.2 ± 122.9	<0.001
	*P* value	0.823	0.001		0.933	0.166	
rs4646155	CC	80.0 ± 91.1	91.5 ± 113.2	0.192	199.1 ± 110.7	245.8 ± 122.3	<0.001
	TT+CT	68.2 ± 74.7	120.6 ± 108.3	0.038	182.1 ± 140.2	227.5 ± 119.6	0.141
	*P* value	0.559	0.048		0.505	0.258	
rs4646188	CC	78.2 ± 85.2	77.8 ± 106.2	0.975	198.0 ± 112.4	259.1 ± 136.1	0.002
	TT+CT	79.2 ± 91.9	101.4 ± 114.1	0.022	197.2 ± 1154.4	238.4 ± 117.6	<0.001
	*P* value	0.948	0.087		0.959	0.164	
rs4830542	CC+CT	81.7 ± 73.4	133.6 ± 125.6	0.013	199.3 ± 174.6	254.6 ± 116.5	0.032
	TT	78.3 ± 93.0	85.7 ± 106.6	0.411	197.0 ± 95.1	239.4 ± 123.3	<0.001
	*P* value	0.823	0.001		0.904	0.298	
rs879922	CC+CG	83.5 ± 75.9	133.7 ± 127.2	0.019	206.4 ± 179.1	259.3 ± 124.5	0.037
	GG	77.9 ± 92.4	78.4 ± 100.5	0.956	195.6 ± 95.1	234.8 ± 120.0	<0.001
	*P* value	0.724	<0.001		0.712	0.058	

## Discussion

EH is rapidly developing into an epidemic and has dramatically increased the global cardiovascular events that have become a serious public health problem. The outlook for EH treatment and prevention is more severe in China [1], especially in minority areas (e.g., the Xinjiang region [26]). The prevalence of EH among adults in Xinjiang is higher than the national average, and there are significant differences in prevalence between different ethnicities, including Han and Uygurs [27]. This study addressed possible relationships between ACE2 polymorphisms and EH in people of the south Xinjiang region of China. We found that certain genotypes of rs2074192 (TT+CT), rs2106809 (TT), rs4240157 (CC+CT), rs4646155 (TT+CT), and rs4830542 (CC+CT) were linked to moderate EH risk (OR = 1.71–1.99), while rs4646188 (TT+CT) and rs879922 (CC+CG) were linked to high EH risk (OR = 3.25–4.86). Our findings are consistent with the observations by Patel et al. [28], who reported ACE2 SNPs (rs2074192, rs4240157, and rs4646188) were associated with higher hypertension risk while rs1978124 was not in a diabetic Australian Caucasian population, and by Benjafield et al. [12], who reported that rs1978124 and rs2285666 were not correlated with EH in Australian persons of Anglo-Celtic descent. With regard to gender (see Supplementary Table 4), we found a high EH risk (OR = 2.24) in males with the rs4646188 TT genotype compared to those with the CC genotype and a higher risk (OR = 6.37) in female carriers of the C allele (TT+CT). However, other ACE2 SNP alleles, rs2074192 (TT), rs2285666 (CC), rs4646142 (GG), and rs4646155 (TT), were only associated with increased EH risk (OR = 1.95–3.47) in males, while rs2106809 (TT), rs4240157 (CC+CT), rs4830542 (CC+CT), and rs879922 (CC+CG) were associated with increased EH risk only in females (OR = 2.56–4.76). SNP rs2285666 (CC) was associated with higher EH risk in northern China (not in northwestern or central China) but only associated with male hypertensive patients in Xinjiang in our study. Our results are in partial agreement with the study by Malard et al. [11], who reported that rs2074192 was correlated with EH in males while rs233575 was correlated in both males and females with European male ancestry. However, SNP rs233575 was related to hypertension in neither males nor females in our study.

The Framingham Study showed that starting from 115/75 mm Hg, the risk for cardiovascular events (e.g., ASCVD) increases following the increase in blood pressure [29]. In this study, we found that two ACE2 SNPs (rs2074192 and rs879922) were associated with moderate and high risk of EH, and both of these loci have also been associated with sudden cardiac arrest [30] but not with the recurrence risk of stroke [31]. Although three SNPs (rs1978124, rs2285666, and rs4646142) were not associated with EH (see Supplementary Table 3), they have exhibited associations with ASCVD (e.g., coronary heart disease [32, 33], ischemic stroke [34]) as well as cardiovascular death [35, 36], suggesting that there is a common genetic basis for hypertension and ASCVD [37]. According to the 2014 NLA recommendations [38], CAS ≥ 50% is defined as a new type of ASCVD. This is based on findings that, in patients with CAS ≥ 50%, the 10-year risk of cardiovascular events is concordant with that of patients suffering from CVD. In our study, we further found that hypertensive patients with the genotype rs2074192 (CC), rs2285666 (TT+CT), or rs4646142 were linked to high risk of CAS ≥ 50% (see Table 3 and Supplementary Table 5). After hypertension, dyslipidemia is the second leading cause of ASCVD [1]. We observed that the lipid spectrum seemed to be in the “normal” range in both the hypertensive and normotensive participants. LDL-C level in the hypertensive group was higher compared with the normotensive group, being even >1.8 mmol/L (the highest target achievement of LDL-C in extremely high-risk EH patients) and was close to level of hypertensive patients in the United States [39]. Compared with EH patients without CAS ≥ 50%, those with CAS ≥ 50% exhibited higher LDL-C and lower HDL-C and were not associated with the three arteriosclerosis risk–related ACE2 SNPs (see Supplementary Table 6).

On the other hand, LAE is often detected in hypertensive patients even before left ventricular hypertrophy (LVH) becomes noticeable, due to increased diastolic or systolic overloading of the left atrium. Indeed, four ACE2 variants that we found to be related to EH risk (rs2074192, rs4240157, rs4646188, and rs879922) have also been correlated with LVH [28, 40]. LAE, unlike LVH, is not used as an indicator of EH-related risk or prognosis in clinical hypertension guidelines, but it is nevertheless an early marker of hypertension-related left ventricular remodeling. We found that not all LVH risk-related ACE2 variation was associated with LAE. SNP rs4240157 was further associated with LAE risk in this study. Beyond that, we newly found that LAD was obviously enlarged in hypertensive patients with the high-EH-risk genotype rs4830542 (CC+CT) or rs4646155 (TT+CT). LAE persists during EH-related heart remodeling, finally leading to AF. In the Framingham Heart Study [41], every 5-mm increase in LAD increased the development of AF by 39%. A recent study correlated rs6632677 with left ventricular remodeling [42], and it has been associated with AF risk in the Chinese population [43]. In contrast, we found that rs6632677 was not associated with risk of AF (see Supplementary Table 7), and the genotypes of the 3 ACE2 SNPs associated with LAE were also associated with increased AF risk (OR = 2.20–2.62).

The activation of RAAS runs through the whole cardiovascular event chain (i.e., atherosclerosis, left atrial remodeling, AF, etc.), and EH is an initiating risk factor for the process [44]. The circulating and tissue RAAS of EH complicated with cardiovascular risk (i.e., dyslipidemia) are excessively activated [45], but genetic ACE2 mutation promotes the development of atherosclerosis and left heart remodeling [46, 47]. SNP rs2074192 has been associated with circulating RAAS activation in hypertensive patients [48], while rs4646155 and rs879922 were not [20]. By contrast, our findings found that not all EH risk-related ACE2 variants (i.e., rs2074192) were associated with the plasma levels of RAAS, not all non-EH risk-related ACE2 variants (i.e., rs4646156) were associated with the plasma levels of RAAS (i.e., renin, see Supplementary Table 8), and the EH risk-related SNP rs879922 was significantly correlated to RAAS activation (i.e., ACE, renin, and ANG I/II). The genotypes (i.e., rs2074192 (CC), rs2285666 (TT+CT), and rs4646142 (CC+CG)) related to high atherosclerosis (CAS ≥ 50%) risk were not associated with high levels of RAAS (see Table 6 and Supplementary Table 8), and the genotypes (i.e., rs42040157 (CC+CT), rs4646155 (TT+CT), and rs4830542 (CC+CT)) associated with LAE were associated with high levels of RAAS (see Table 6), suggesting that ACE2 genetic variants have the potential to affect circulating RAAS activation.

Some limitations should be mentioned. First, since our sample size was not large enough, further prospective large sample studies are needed to validate our findings. Second, the possibility of false-positive findings should be considered, especially for secondary studies based on our results.

In conclusion, the ACE2 variant rs2074192 was associated with EH and EH with CAS ≥ 50%, while three ACE2 variants (rs4240157, rs4646155, and rs4830542) were associated with EH- and hypertension-related AF and left atrial remodeling. Specially, our data show for the first time that the ACE2 SNP rs4830542 had those effects, while rs2048683 did not, suggesting that the ACE2 SNP rs4830542 may be a novel genetic susceptibility marker for EH (especially in females) and EH-related cardiovascular events. Our observations further support that the genetic predisposition of ACE2 SNPs associated with the risk of EH and EH with cardiovascular risk factors and events warrants large-scale evaluation in the south Xinjiang region.

## Supplementary Information

Supplementary Information
