# Supplementary material for: Association of ACE2 genetic polymorphisms with hypertension-related target organ damages in south Xinjiang
Source: Hypertens Res. 2018 Dec 12;42(5):681–9. doi: 10.1038/s41440-018-0166-6 (PMC6477792; doi:10.1038/s41440-018-0166-6)
Supplement: Supplementary file 1 — Supplementary Information [file 41440_2018_166_MOESM1_ESM.doc]

**Supplementary Information**

**Result**

**ACE2 SNPs and genotype frequencies**

Because ACE2 is on the X-chromosome, we assessed Hardy-Weinberg equilibrium only for female subjects. Not all ACE2 SNPs examined were in Hardy-Weinberg equilibrium (P>0.05) and heterogeneity was observed in study participants (see Supplementary Table 2). In female subjects ACE2 SNP rs2074192, rs2285666, rs4240157, rs4646142, rs4830542 and rs879922 were in Hardy-Weinberg equilibrium (all P>0.05).

**Association of ACE2 SNPs with EH**

As shown in Supplementary Table 3, 7 ACE2 SNP (i.e., rs1978124, rs2048683, rs2285666, rs233575, rs4646142, rs4646156, and rs6632677) were not associated with EH (all P>0.05).

**Association of ACE2 SNPs with EH complicated by CAS ≥50%**

As shown in Supplementary Table 5, 11 ACE2 SNPs (i.e., rs1978124, rs2048683, rs2106809, rs233575, rs4240157, rs4646155, rs4646156, rs4646188, rs4830542, rs6632677 and rs879922) were not associated with EH complicated by CAS ≥50%(all P>0.05).

**Association of ACE2 SNPs with dyslipidemia**

As shown in Supplementary Table 6, significant differences were observed between hypertensive and normotensive participants on the levels of LDL-C and HDL-C (all P=0.001 or <0.001) with the exception of TRIG, CHOL and Lp(a) (all P>0.05), and no significance were observed between the high arteriosclerosis risk and control genotype of arteriosclerosis risk related ACE2 SNPs (i.e., rs2074192, rs2285666 and rs4646142) on the levels of TRIG, CHOL, LDL-C HDL-C and Lp(a) (all P>0.05).

**Association of ACE2 SNPs with EH complicated by AF**

As shown in Supplementary Table 7, 9 ACE2 SNPs (i.e., rs1978124, rs2048683, rs2074192, rs2106809, rs233575, rs4646156, rs4646188, rs6632677 and rs879922) were not associated with EH complicated by AF(all P>0.05).

**Association of ACE2 SNPs with RAAS**

As shown in Supplementary Table 8, 2 ACE2 SNPs (rs2285666 and rs4646142) were not associated with RAAS levels (all P>0.05) while SNP rs4646156 was linked to RAAS activation (i.e, renin, P=0.004).

**Table legends**

Supplementary Table 1 ACE2 SNP primers used in the Sequenom MassARRAY system

Supplementary Table 2 Descriptive information on ACE2 SNPs in study participants

Supplementary Table 3 Association of 7 ACE2 SNPs with EH in participants

Supplementary Table 4 Association of ACE2 SNPs with EH in male and female participants

Supplementary Table 5 Association of 11 ACE2 SNPs with EH complicated by CAS ≥50% in hypertensive participants

Supplementary Table 6 Association of arteriosclerosis risk related ACE2 SNPs and dyslipidemia in hypertensive participants

Supplementary Table 7 Association of 9 ACE2 SNPs with EH complicated by AF in hypertensive participants

Supplementary Table 8 Association of ACE2 SNPs with the RAAS activity in participants

Supplementary Table 1 The primers of ACE2 SNPs in the Sequenom MassARRAY system

| **NQ.** | **SNP_ID** | **2nd-** **reverse PCR primer (5’-3’)** | **1st-** **forward PCR primer (5’-3’)** | **extension primer (5’-3’)** |
| --- | --- | --- | --- | --- |
| 1 | *rs1978124* | *ACGTTGGATGGAGAGAACTTTGGAAACCTG* | *ACGTTGGATGAAGCTGCTGATGTAGAAGTG* | *CCATATCTCTATCTGATGGAC* |
| 2 | *rs2048683* | *ACGTTGGATGCAGTGAACATGGCAGTGTAG* | *ACGTTGGATGTGATCCAGCAATCCCTCTTC* | *GGCAGTGTAGATATCTTTATGAAG* |
| 3 | *rs2074192* | *ACGTTGGATGTTAGGTTCATCAACAGCTCC* | *ACGTTGGATGCCCTTAAACACAGCAGTCAC* | *CAAGGGTGGAAATGTATAAATGGTTGG* |
| 4 | *rs2106809* | *ACGTTGGATGAATAACAGTCTCTCTCCCCC* | *ACGTTGGATGTTCCTGGGTAGATGGCAATG* | *GTTTTCCTGCACACCATCTGAT* |
| 5 | *rs2285666* | *ACGTTGGATGCTGAGAGAAAAGTAAATTTCA* | *ACGTTGGATGCCAGATAATCCACAAGAATGC* | *CATAATCACTACTAAAAATTAGTAGC* |
| 6 | *rs233575* | *ACGTTGGATGAGGTCCTATGACCAAGTCTC* | *ACGTTGGATGTTTCTTATGTGCCTCCCCAG* | *TCCTATGACCAAGTCTCTATAGTA* |
| 7 | *rs4240157* | *ACGTTGGATGTTGCTCAGTGAATTGGCCTC* | *ACGTTGGATGTTTCCATGCAGTGAGGGTTG* | *CTCAGAACATTACAGAATCAAAC* |
| 8 | *rs4646142* | *ACGTTGGATGTTAGGTGAAGCGTTCCTGTC* | *ACGTTGGATGAGCCCATGGACCCAATAAAG* | *GGAGATGGTTGCTTTGTAGTCTC* |
| 9 | *rs4646155* | *ACGTTGGATGGGCATGTTCTTAACCTTGGC* | *ACGTTGGATGCCAATATGACCCTGTAAACC* | *GGATGAACCTTGGCAAAATAAACTT* |
| 10 | *rs4646156* | *ACGTTGGATGGGGAAATAGATATGATGGGC* | *ACGTTGGATGCCTTAGGTACTTGGACCTTC* | *GGGCCATGGAACAGG* |
| 11 | *rs4646188* | *ACGTTGGATGGATATTCTACTCAGAAACG* | *ACGTTGGATGCTCTGTGTTCCCTTCTGTTG* | *GGGGAACGTAGAATTTTAGTTGAATG* |
| 12 | *rs4830542* | *ACGTTGGATGAGGATGAGCTCATGCACAAG* | *ACGTTGGATGCAAAAATAAGAAGAAGAAAGG* | *TCTTTCTGGTCTCCCTCT* |
| 13 | *rs6632677* | *ACGTTGGATGAGCCTCAGAAGAGACCATAG* | *ACGTTGGATGAGTTCAGCTGGATCTTCTGC* | *CTCTACCATAGCTCTAGCCA* |
| 14 | *rs879922* | *ACGTTGGATGGCTCCAGCAAATTCAAGGAC* | *ACGTTGGATGGGCAGTTTATTGTACATTGTG* | *CTCAAGGACTGGGGTTA* |

Supplementary Table 2 Descriptive information on ACE2 SNPs in study participants

| **NQ** | **ACE2 SNPs** | **MAF in**  **CHB/CEU*** | **Major/minor allele** | **MAF** | | **Power** | ***P_HWE_*-value^#^** |
| --- | --- | --- | --- | --- | --- | --- | --- |
|  |  |  |  | **Normotensive** | **Hypertensive** |  |  |
| 1 | *rs1978124* | 0.006/0.483 | *C/T* | 0.135 | 0.108 | 0.945 | 0.003 |
| 2 | *rs2048683* | 0.006/0.409 | *G/T* | 0.113 | 0.067 | 0.807 | 0.003 |
| 3 | *rs2074192* | 0.463/0.349 | *C/T* | 0.380 | 0.434 | 0.999 | 0.659 |
| 4 | *rs2106809* | 0.481/0.262 | *T/C* | 0.376 | 0.332 | 0.999 | 0.011 |
| 5 | *rs2285666* | 0.488/0.262 | *T/C* | 0.489 | 0.488 | 0.999 | 0.303 |
| 6 | *rs233575* | 0.006/0.362 | *T/C* | 0.119 | 0.071 | 0.859 | 0.006 |
| 7 | *rs4240157* | 0.044/0.383 | *T/C* | 0.146 | 0.133 | 0.973 | 0.456 |
| 8 | *rs4646142* | 0.488/0.261 | *C/G* | 0.485 | 0.485 | 0.999 | 0.216 |
| 9 | *rs4646155* | 0.038/0.000 | *C/T* | 0.069 | 0.162 | 0.172 | <0.001 |
| 10 | *rs4646156* | 0.006/0.403 | *T/A* | 0.097 | 0.071 | 0.607 | 0.001 |
| 11 | *rs4646188* | 0.000/0.087 | *T/C* | 0.294 | 0.267 | 0.999 | <0.001 |
| 12 | *rs4830542* | 0.044/0.383 | *T/C* | 0.146 | 0.131 | 0.973 | 0.380 |
| 13 | *rs6632677* | 0.094/0.007 | *G/C* | 0.043 | 0.060 | 0.070 | 0.016 |
| 14 | *rs879922* | 0.044/0.389 | *G/C* | 0.139 | 0.182 | 0.958 | 0.146 |

*MAF: minor allele frequency; CHB：Han Chinese in Beijing, China；CEU：Utah residents with Northern and Western European ancestry

# *P*_HWE_ value for female participants

Supplementary Table 3 Association of 7 ACE2 SNPs with EH in participants

| **ACE2 SNPs** | | **Normotensive**  **(N/%)** | **Hypertensive**  **(N/%)** | **OR(95%CI)*** | ***P*-value** |
| --- | --- | --- | --- | --- | --- |
| *rs1978124* | *CC* | 191(82.0) | 336(83.6) | 1.00 |  |
|  | *TT+CT* | 42(18.0) | 66(16.4) | 0.98(0.55-1.73) | 0.939 |
| *rs2048683* | *GG* | 203(87.1) | 357(88.8) | 1.00 |  |
|  | *TT+GT* | 30(12.9) | 45(11.2) | 0.85(0.46-1.58) | 0.601 |
| *rs2285666* | *CC* | 85(36.5) | 146(36.3) | 1.05(0.70-1.57) | 0.829 |
|  | *TT+CT* | 148(63.5) | 256(63.7) | 1.00 |  |
| *rs233575* | *CC+CT* | 36(15.5) | 48(11.9) | 1.00 |  |
|  | *TT* | 197(84.5) | 354(88.1) | 1.31(0.70-2.45) | 0.398 |
| *rs4646142* | *CC+CG* | 149(63.9) | 254(63.2) | 1.00 |  |
|  | *GG* | 84(36.1) | 148(36.8) | 1.07(0.72-1.60) | 0.736 |
| *rs4646156* | *AA+AT* | 30(12.9) | 48(11.9) | 1.00 |  |
|  | *TT* | 203(87.1) | 354(88.1) | 1.13(0.61-2.09) | 0.704 |
| *rs6632677* | *CC+CG* | 15(6.4) | 34(8.5) | 1.00 |  |
|  | *GG* | 218(93.6) | 368(91.5) | 0.79(0.39-1.58) | 0.502 |

* After adjustment for nationality, gender, age, smoking, BMI, TRIG, LDL-C, HDL-C, Lp(a), FBS, UA, HsCRP and Ang II

Supplementary Table 4 Association of ACE2 SNPs with EH in male and female participants

| **ACE2 SNPs** | **Male** | | | | |  | **Female** | | | | |
| --- | --- | --- | --- | --- | --- | --- | --- | --- | --- | --- | --- |
|  | **Genotypes** | **Normotensive**  **(N/%)** | **Hypertensive**  **(N/%)** | **OR(95%CI)*** | ***P-value*** |  | **Genotypes** | **Normotensive**  **(N/%)** | **Hypertensive**  **(N/%)** | **OR(95%CI)*** | ***P-value*** |
| *rs1978124* | *CC* | 84(90.3) | 151(91.0) | 1.00 |  |  | *CC* | 107(76.4) | 185(78.4) | 1.00 |  |
|  | *TT* | 9(9.3) | 15(9.0) | 0.47(0.15-1.47) | 0.191 |  | *TT+CT* | 33(23.6) | 51(21.6) | 1.49(0.75-2.94) | 0.257 |
| *rs2048683* | *GG* | 87(93.5) | 157(94.6) | 1.00 |  |  | *GG* | 116(82.9) | 200(84.7) | 1.00 |  |
|  | *TT* | 6(6.5) | 9(5.4) | 0.43(0.11-1.61) | 0.210 |  | *TT+GT* | 24(17.1) | 36(15.3) | 1.12(0.54-2.32) | 0.754 |
| *rs2074192* | *CC* | 64(68.8) | 81(48.8) | 1.00 |  |  | *CC* | 54(38.6) | 81(34.3) | 1.00 |  |
|  | *TT* | 64(68.8) | 81(48.8) | 2.43(1.29-4.58) | 0.006 |  | *TT+CT* | 86(61.4) | 155(65.7) | 1.48(0.87-2.51) | 0.145 |
| *rs2106809* | *CC* | 33(35.5) | 54(32.5) | 1.00 |  |  | *CC+CT* | 88(62.9) | 121(51.3) | 1.00 |  |
|  | *TT* | 60(64.5) | 112(67.5) | 1.24(0.64-2.39) | 0.522 |  | *TT* | 52(37.1) | 115(48.7) | 2.56(1.43-4.60) | 0.002 |
| *rs2285666* | *CC* | 38(40.9) | 96(57.8) | 1.95(1.05-3.63) | 0.035 |  | *CC* | 47(33.6) | 50(21.2) | 0.63(0.35-1.12) | 0.116 |
|  | *TT* | 55(59.1) | 70(42.2) | 1.00 |  |  | *TT+CT* | 93(66.4) | 185(78.8) | 1.00 |  |
| *rs233575* | *CC* | 9(9.7) | 6(3.6) | 1.00 |  |  | *CC+CT* | 27(19.3) | 42(17.8) | 1.00 |  |
|  | *TT* | 84(90.3) | 160(96.4) | 2.19(0.53-9.06) | 0.278 |  | *TT* | 113(80.7) | 194(82.2) | 1.01(0.48-2.13) | 0.985 |
| *rs4240157* | *CC* | 14(15.1) | 15(9.0) | 0.45(0.15-1.29) | 0.135 |  | *CC+CT* | 29(20.7) | 77(32.6) | 4.63(2.25-9.52) | <0.001 |
|  | *TT* | 79(84.9) | 151(91.0) | 1.00 |  |  | *TT* | 111(79.3) | 159(67.4) | 1.00 |  |
| *rs4646142* | *CC* | 56(60.2) | 70(42.2) | 1.00 |  |  | *CC+CG* | 93(66.4) | 184(78.0) | 1.00 |  |
|  | *GG* | 37(39.8) | 96(57.8) | 2.01(1.08-3.74) | 0.027 |  | *GG* | 47(33.6) | 52(22.0) | 0.67(0.38-1.20) | 0.178 |
| *rs4646155* | *CC* | 88(94.6) | 140(84.3) | 1.00 |  |  | *CC* | 123(87.9) | 194(82.2) | 1.00 |  |
|  | *TT* | 5(5.4) | 26(15.7) | 3.47(1.08-11.16) | 0.037 |  | *TT+CT* | 17(12.1) | 42(17.8) | 0.92(0.42-2.02) | 0.837 |
| *rs4646156* | *AA* | 6(6.5) | 9(5.4) | 1.00 |  |  | *AA+AT* | 24(17.1) | 39(16.5) | 1.00 |  |
|  | *TT* | 87(93.5) | 157(94.6) | 2.33(0.62-8.79) | 0.210 |  | *TT* | 116(82.9) | 197(83.5) | 0.82(0.40-1.68) | 0.582 |
| *rs4646188* | *CC* | 51(54.8) | 73(44.0) | 1.00 |  |  | *CC* | 23(16.4) | 12(5.1) | 1.00 |  |
|  | *TT* | 42(45.2) | 93(56.0) | 2.24(1.10-4.53) | 0.026 |  | *TT+CT* | 117(83.6) | 224(94.9) | 6.37(2.50-16.28) | <0.001 |
| *rs4830542* | *CC* | 14(15.1) | 15(9.0) | 0.42(0.14-1.22) | 0.111 |  | *CC* | 29(20.7) | 75(31.8) | 4.76(2.32-9.78) | <0.001 |
|  | *TT* | 79(84.9) | 151(91.0) | 1.00 |  |  | *TT+CT* | 111(79.3) | 161(68.2) | 1.00 |  |
| *rs6632677* | *CC* | 2(2.2) | 14(8.4) | 1.00 |  |  | *CC+CG* | 13(9.3) | 20(8.5) | 1.00 |  |
|  | *GG* | 91(97.8) | 152(91.6) | 0.21(0.04-1.05) | 0.057 |  | *GG* | 127(90.7) | 216(91.5) | 1.34(0.58-3.13) | 0.495 |
| *rs879922* | *CC* | 14(15.1) | 15(9.0) | 0.42(0.15-1.14) | 0.088 |  | *CC+CG* | 26(18.6) | 116(49.2) | 4.71(2.58-8.58) | <0.001 |
|  | *GG* | 79(84.9) | 151(91.0) | 1.00 |  |  | *GG* | 114(81.4) | 120(50.8) | 1.00 |  |

* After adjustment for nationality, gender, age, smoking, BMI, TRIG, LDL-C, HDL-C, Lp(a), FBS , UA, HsCRP and Ang II.

Supplementary Table 5 Association of 11 ACE2 SNPs with EH complicated by CAS ≥50% in hypertensive participants

| **ACE2 SNPs** | | **Non-CAS≥50%**  **(N/%)** | **CAS≥50% (N/%)** | **OR(95%CI)*** | ***P*-value** |
| --- | --- | --- | --- | --- | --- |
| *rs1978124* | *CC* | 285(84.1) | 51(81.0) | 1.00 |  |
|  | *TT+CT* | 54(15.9) | 12(19.0) | 1.08(0.45-2.61) | 0.857 |
| *rs2048683* | *GG* | 306(90.3) | 51(81.0) | 1.00 |  |
|  | *TT+GT* | 33(9.0) | 12(19.0) | 1.96(0.79-4.84) | 0.146 |
| *rs2106809* | *CC+CT* | 144(42.5) | 31(49.2) | 1.00 |  |
|  | *TT* | 195(57.5) | 32(50.8) | 0.53(0.26-1.06) | 0.071 |
| *rs233575* | *CC+CT* | 39(11.5) | 9(14.3) | 1.00 |  |
|  | *TT* | 300(88.5) | 54(85.7) | 1.07(0.41-2.77) | 0.891 |
| *rs4240157* | *CC+CT* | 74(21.8) | 18(28.6) | 1.14(0.52-2.52) | 0.747 |
|  | *TT* | 265(78.2) | 45(71.4) | 1.00 |  |
| *rs4646155* | *CC+CT* | 285(84.1) | 55(87.3) | 1.24(0.50-3.09) | 0.645 |
|  | *TT* | 54(15.9) | 8(12.7) | 1.00 |  |
| *rs4646155* | *CC* | 279(82.3) | 55(87.3) | 1.37(0.56-3.38) | 0.493 |
|  | *TT+CT* | 60(17.7) | 8(12.7) | 1.00 |  |
| *rs4646156* | *AA+AT* | 36(10.6) | 12(19.0) | 1.00 |  |
|  | *TT* | 303(89.4) | 51(81.0) | 0.68(0.28-1.67) | 0.398 |
| *rs4646188* | *CC* | 74(21.8) | 11(17.5) | 1.00 |  |
|  | *TT+CT* | 265(78.2) | 52(82.5) | 0.98(0.41-2.34) | 0.956 |
| *rs4830542* | *CC+CT* | 72(21.2) | 18(28.6) | 1.21(0.54-2.74) | 0.647 |
|  | *TT* | 267(78.8) | 45(71.4) | 1.00 |  |
| *rs6632677* | *CC+CG* | 31(9.1) | 3(4.8) | 1.00 |  |
|  | *GG* | 308(90.9) | 60(95.2) | 1.88(0.0.52-6.77) | 0.337 |
| *rs879922* | *CC+CG* | 104(30.7) | 27(42.9) | 1.52(0.55-4.25) | 0.424 |
|  | *GG* | 235(69.3) | 36(57.1) | 1.00 |  |

* After adjustment for nationality, gender, age, smoking, BMI, LDL-C, HDL-C, FBS, UA, HsCRP and Ang II

Supplementary Table 6 Association of arteriosclerosis risk related ACE2 SNPs and dyslipidemia in hypertensive participants

| **ACE2 SNPs** | | ***rs2074192*** | | |  | ***rs2285666*** | | |  | ***rs4646142*** | | |
| --- | --- | --- | --- | --- | --- | --- | --- | --- | --- | --- | --- | --- |
|  |  | **CC** | **TT+CT** | ***P-value*** |  | **CC** | **TT+CT** | ***P-value*** |  | **CC+CG** | **GG** | ***P-value*** |
| TRIG | Non CAS≥50% | 1.29±0.61 | 1.37±0.59 | 0.219 |  | 1.29±0.61 | 1.38±0.60 | 0.214 |  | 1.37±0.60 | 1.30±0.60 | 0.234 |
|  | CAS≥50% | 1.28±0.75 | 1.60±0.82 | 0.116 |  | 1.48±0.91 | 1.39±0.76 | 0.718 |  | 1.39±0.76 | 1.48±0.91 | 0.718 |
|  | *P-value* | 0.925 | 0.184 |  |  | 0.445 | 0.868 |  |  | 0.835 | 0.453 |  |
| CHOL | Non CAS≥50% | 4.57±1.11 | 4.66±1.29 | 0.468 |  | 4.49±1.25 | 4.72±1.20 | 0.092 |  | 4.73±1.20 | 4.47±1.24 | 0.060 |
|  | CAS≥50% | 4.92±1.03 | 4.99±1.13 | 0.819 |  | 4.66±1.24 | 5.04±1.01 | 0.233 |  | 5.04±1.01 | 4.66±1.24 | 0.233 |
|  | *P-value* | 0.086 | 0.214 |  |  | 0.605 | 0.084 |  |  | 0.095 | 0.575 |  |
| LDL-C | Non CAS≥50% | 2.72±0.70 | 2.85±0.83 | 0.161 |  | 2.82±0.77 | 2.79±0.79 | 0.713 |  | 2.80±0.79 | 2.80±0.78 | 0.920 |
|  | CAS≥50% | 3.32±0.84 | 2.93±0.81 | 0.070 |  | 3.42±0.96 | 3.07±0.80 | 0.161 |  | 3.07±0.80 | 3.42±0.96 | 0.161 |
|  | *P-value* | <0.001 | 0.621 |  |  | 0.006 | 0.028 |  |  | 0.034 | 0.005 |  |
| HDL-C | Non CAS≥50% | 1.33±0.25 | 1.28±0.27 | 0.375 |  | 1.27±0.09 | 1.32±0.29 | 0.262 |  | 1.32±0.29 | 1.27±0.09 | 0.262 |
|  | CAS≥50% | 1.15±0.25 | 1.19±0.30 | 0.188 |  | 1.17±0.22 | 1.18±0.31 | 0.796 |  | 1.18±0.31 | 1.17±0.22 | 0.636 |
|  | *P-value* | <0.001 | 0.167 |  |  | 0.004 | 0.004 |  |  | 0.005 | 0.003 |  |
| Lp(a) | Non CAS≥50% | 0.23±0.20 | 0.25±0.22 | 0.487 |  | 0.25±0.22 | 0.24±0.21 | 0.583 |  | 0.23±0.20 | 0.26±0.23 | 0.225 |
|  | CAS≥50% | 0.22±0.20 | 0.28±0.21 | 0.245 |  | 0.21±0.19 | 0.26±0.20 | 0.455 |  | 0.26±0.21 | 0.21±0.19 | 0.455 |
|  | *P-value* | 0.738 | 0.478 |  |  | 0.501 | 0.574 |  |  | 0.438 | 0.427 |  |

Supplementary Table 7 Association of 9 ACE2 SNPs with EH complicated by AF in hypertensive participants

| **ACE2 SNPs** | | **Non-AF**  **(N/%)** | **AF**  **(N/%)** | **OR(95%CI)*** | ***P*-value** |
| --- | --- | --- | --- | --- | --- |
| *rs1978124* | *CC* | 272(83.4) | 64(84.2) | 1.00 |  |
|  | *TT+CT* | 54(16.6) | 12(15.8) | 0.55(0.23-1.31) | 0.178 |
| *rs2048683* | *GG* | 287(88.0) | 70(92.1) | 1.00 |  |
|  | *TT+GT* | 39(12.0) | 6(7.9) | 0.45(0.16-1.24) | 0.121 |
| *rs2074192* | *CC* | 131(40.2) | 31(40.8) | 0.98(0.56-1.73) | 0.954 |
|  | *TT+CT* | 195(59.8) | 45(59.2) | 1.00 |  |
| *rs2106809* | *CC+CT* | 147(45.1) | 28(36.8) | 1.00 |  |
|  | *TT* | 179(54.9) | 48(63.2) | 0.89(0.46-1.72) | 0.725 |
| *rs233575* | *CC+CT* | 39(12.0) | 9(11.8) | 1.00 |  |
|  | *TT* | 287(88.0) | 67(88.2) | 1.14(0.46-2.83) | 0.782 |
| *rs4646156* | *AA+AT* | 42(12.9) | 6(7.9) | 1.00 |  |
|  | *TT* | 284(87.1) | 70(92.1) | 2.69(0.98-7.40) | 0.056 |
| *rs4646188* | *CC* | 64(19.6) | 21(27.6) | 1.00 |  |
|  | *TT+CT* | 262(80.4) | 55(72.4) | 0.58(0.28-1.20) | 0.140 |
| *rs6632677* | *CC+CG* | 29(8.9) | 5(6.6) | 1.00 |  |
|  | *GG* | 297(91.1) | 71(93.4) | 1.30(0.46-3.68) | 0.628 |
| *rs879922* | *CC+CG* | 101(31.0) | 30(39.5) | 1.16(0.54-2.46) | 0.709 |
|  | *GG* | 225(69.0) | 46(60.5) | 1.00 |  |

* After adjustment for nationality, gender, age, smoking, BMI, SBP, DBP, LDL-C, HDL-C, FBS, UA, HsCRP, Ang II and LAD.

Supplementary Table 8 Association of ACE2 SNPs with the RAAS activity in participants

| **RAAS** | | ***rs2285666*** | | |  | ***rs4646142*** | | |  | ***rs4646156*** | | |
| --- | --- | --- | --- | --- | --- | --- | --- | --- | --- | --- | --- | --- |
|  |  | ***CC*** | ***TT+CT*** | ***P-value*** |  | ***CC+CG*** | ***GG*** | ***P-value*** |  | ***AA+AT*** | ***TT*** | ***P-value*** |
| **ACE**  **(U/L)** | Normotensive | 36.5±16.5 | 35.1±20.7 | 0.559 |  | 35.7±21.2 | 35.4±15.1 | 0.882 |  | 42.9±15.4 | 49.2±15.2 | 0.077 |
|  | Hypertensive | 42.4±15.5 | 41.8±30.0 | 0.804 |  | 41.5±30.0 | 42.8±15.9 | 0.561 |  | 34.5±19.5 | 41.0±26.6 | 0.003 |
|  | *P-value* | 0.007 | 0.016 |  |  | 0.039 | 0.001 |  |  | 0.026 | 0.037 |  |
| **Renin**  **(pg/mL)** | Normotensive | 25.5±25.4 | 24.0±29.6 | 0.705 |  | 23.8±29.6 | 25.9±25.2 | 0.591 |  | 21.0±18.4 | 52.4±35.0 | <0.001 |
|  | Hypertensive | 38.3±32.2 | 32.6±33.8 | 0.100 |  | 32.8±33.9 | 37.8±32.2 | 0.150 |  | 25.1±29.2 | 32.3±32.4 | 0.009 |
|  | *P-value* | 0.001 | 0.011 |  |  | 0.007 | 0.002 |  |  | 0.457 | <0.001 |  |
| **Ang I**  **(ng/L)** | Normotensive | 1.96±1.37 | 2.13±1.48 | 0.402 |  | 2.12±1.48 | 1.97±1.38 | 0.422 |  | 1.81±1.43 | 3.08±1.75 | 0.001 |
|  | Hypertensive | 2.76±1.91 | 2.66±1.69 | 0.582 |  | 2.66±1.69 | 2.76±1.90 | 0.597 |  | 2.10±1.44 | 2.65±1.77 | <0.001 |
|  | *P-value* | <0.001 | 0.001 |  |  | 0.001 | <0.001 |  |  | 0.298 | 0.106 |  |
| **Ang II**  **(ng/L)** | Normotensive | 76.7±86.2 | 80.1±91.8 | 0.783 |  | 80.0±91.5 | 76.8±86.8 | 0.795 |  | 95.0±82.3 | 131.8±128.5 | 0.127 |
|  | Hypertensive | 110.8±125.0 | 88.3±104.5 | 0.067 |  | 88.6±104.9 | 109.9±124.3 | 0.067 |  | 76.5±90.6 | 91.6±109.8 | 0.080 |
|  | *P-value* | 0.016 | 0.431 |  |  | 0.410 | 0.018 |  |  | 0.293 | 0.020 |  |
| **ALD**  **(ng/L)** | Normotensive | 191.6±132.1 | 200.9±101.7 | 0.549 |  | 201.9±100.6 | 189.5±133.8 | 0.423 |  | 211.0±172.7 | 279.1±133.7 | 0.054 |
|  | Hypertensive | 256.8±125.6 | 234.7±119.2 | 0.080 |  | 235.8±119.1 | 254.7±126.1 | 0.134 |  | 195.5±102.4 | 237.8±119.5 | <0.001 |
|  | *P-value* | <0.001 | 0.003 |  |  | 0.002 | <0.001 |  |  | 0.634 | 0.028 |  |
